# E-Health: a novel way to redesigning healthcare

**DOI:** 10.1007/s12471-016-0843-5

**Published:** 2016-05-10

**Authors:** E. E. van der Wall

**Affiliations:** Holland Heart House, Netherlands Society of Cardiology, Utrecht, The Netherlands

Electronic healthcare (e-health) will significantly alter the way physicians practise medicine [1, 2]. Electronic medical records with specialised software programs can increase the quality of patient care, reduce unnecessary medical tests, and directly connect with pharmacies to transmit prescriptions. Electronic communication allows physicians to respond to patients’ clinical concerns and questions, and Internet access can provide physicians better access to literature. One of those e‑health aspects is remote patient care, which has made considerable progress in remote monitoring of ICD patients [3–7]. Nevertheless, there is significant physician hesitance about implementing medical computerisation: patient email can potentially overload physicians with additional work, websites can direct patients to poor medical information, the computerised interface can degrade the patient-physician relationship, and health regulations can create concern over electronic privacy issues. The finances of e‑health appear promising, yet conflicting studies create uncertainty. However, if managed appropriately, the potential disadvantages of e‑health can be minimised, and the benefits of e‑health in clinical practices can be obtained [1].

In the present issue of the Netherlands Heart Journal, Treskes et al. [8] extensively discuss the current status of health-monitoring devices and the implementation of individualised healthcare services. The authors state that the consumer market for personal healthcare devices is developing rapidly and with the current healthcare-related investments by technological companies it can be expected that the way healthcare is provided will change dramatically. In this way, healthcare will be redesigned with the final aim to connect smart technology in order to improve patient outcome. Although a variety of initiatives under the banner of e‑health are deployed, most are characterised by either industry-driven developments without proven clinical effectiveness or individual initiatives lacking the embedding within the traditional organisations. However, the introduction of numerous smart devices and internet-based technologies facilitates the fundamental re-design of healthcare based on the principle of achieving best possible care for the individual patient at the lowest possible costs. Hospitals are still willing to invest enormous amounts of money and human resources in generic mainstream systems, whereas it can be expected that in the near future information will be stored in the cloud and networked distributed applications will provide optimal support for treating individual patients [9]. A simple calculation of costs returns an astonishing 2.4 billion euros spent by approximately 80 Dutch hospitals to introduce a basic hospital information system [8].

According to the authors, several hurdles should be taken to have this achieved [8]. First, instead of reimbursing individual healthcare providers, it will be necessary to reimburse healthcare systems. Second, it is important to recognise possible hesitations by the involved stakeholders. Third, it is important that data safety is ensured and monitored by an independent inspectorate. Lastly, it is vital that patients are able to refuse the exchange of their data between healthcare providers and that access rights are well described. Of course, the privacy of the patient has to be guaranteed.

Along those lines, the European Society of Cardiology (ESC) has recently published a Position Statement on e‑health in the January 2016 issue of the European Heart Journal [10]. The ESC is very convinced about the great value of e‑health, but at the same time very much aware that there are important societal and professional constraints that reduce the impact of such innovation, including legal, ethical, and data protection issues. Healthcare professionals may be resistant to such innovation, particularly if the technologies are considered to be solutions seeking a problem and where the evidence for the impact on quality of care is seen as less than robust. Regulatory bodies, reimbursement authorities, and national and international political bodies often find it difficult to react quickly, or consistently, to this rapidly changing area.

The vision of the ESC is to play a pro-active role in all aspects of the e‑health agenda, helping to develop, assess, and implement effective ICT innovations in the support of cardiovascular health and health-related activity across Europe. The action plan of the ESC includes the following issues: 1) to facilitate wider implementation of e‑health, 2) to educate and train ESC members in the appropriate use of e‑health, 3) to play an active role in discussions regarding regulation and quality control of ICT technologies, 4) to play an active role in the societal and political discussions regarding data security and confidentiality issues, recognising the geographical variation in legal constraints, 5) to support research into the development, evaluation, and implementation of e‑health technologies, 6) to promote policy dialogue related to e‑health at local, national, and international level with all relevant stakeholders, including payers, and 7) to provide a resource for citizens in the member countries to assist them in assessing the potential benefit and risk of e‑health applications in cardiovascular disease prevention, diagnosis, and treatment. With this ESC action plan, e‑health can offer innovate solutions to health issues, being a key enabling technology to improve care to those patients living with chronic conditions, particularly at a time of constrained healthcare funding.

At our national level, the Netherlands Society of Cardiology (NVVC) has recently installed a Project Group E‑Health with the aim to investigate how e‑health can be implemented in our daily healthcare for patients with cardiovascular diseases, for instance in those with heart failure [11, 12].

To summarise the addressed e‑health issues, Treskes et al. [8] validly conclude that the question is not whether e‑healthcare will change our patient management, but to what extent healthcare professionals together with patients will be able to fundamentally redesign healthcare in a structured manner. This process should start with defining the needs of patients based on the principle of best achievable care at the lowest possible costs.
